# The Wilms Tumor Gene *wt1a* Contributes to Blood-Cerebrospinal Fluid Barrier Function in Zebrafish

**DOI:** 10.3389/fcell.2021.809962

**Published:** 2022-01-11

**Authors:** Vera L. Hopfenmüller, Birgit Perner, Hanna Reuter, Thomas J. D. Bates, Andreas Große, Christoph Englert

**Affiliations:** ^1^ Leibniz Institute on Aging - Fritz Lipmann Institute (FLI), Jena, Germany; ^2^ Institute of Biochemistry and Biophysics, Friedrich-Schiller-University Jena, Jena, Germany

**Keywords:** Wilms tumor protein, zebrafish, choroid plexus, CRISPR/cas9, dye accumulation assay, fluorescent tracer

## Abstract

The Wilms tumor suppressor gene *Wt1* encodes a zinc finger transcription factor, which is highly conserved among vertebrates. It is a key regulator of urogenital development and homeostasis but also plays a role in other organs including the spleen and the heart. More recently additional functions for Wt1 in the mammalian central nervous system have been described. In contrast to mammals, bony fish possess two paralogous *Wt1* genes, namely *wt1a* and *wt1b.* By performing detailed *in situ* hybridization analyses during zebrafish development, we discovered new expression domains for *wt1a* in the dorsal hindbrain, the caudal medulla and the spinal cord. Marker analysis identified *wt1a* expressing cells of the dorsal hindbrain as ependymal cells of the choroid plexus in the myelencephalic ventricle. The choroid plexus acts as a blood-cerebrospinal fluid barrier and thus is crucial for brain homeostasis. By employing *wt1a* mutant larvae and a dye accumulation assay with fluorescent tracers we demonstrate that Wt1a is required for proper choroid plexus formation and function. Thus, Wt1a contributes to the barrier properties of the choroid plexus in zebrafish, revealing an unexpected role for Wt1 in the zebrafish brain.

## Introduction

The choroid plexus (CP) is a cellular structure within each ventricle of the vertebrate brain. It constitutes a barrier between the blood and the cerebrospinal fluid (CSF) and consists of epithelial cells, fenestrated blood vessels, stromal cells and extracellular matrix (Wolburg and Paulus, 2010). The cuboid epithelial cells of the CP, called ependymal cells are connected by tight junctions and are highly polarized. They harbor cilia and microvilli on their apical surface that projects into the ventricle and face the Soma with their basal side (Lun et al., 2015). The ependymal cells of the CP derive from neuroepithelial cells. The respective specification requires repression of neural cell fate (Imayoshi et al., 2008) and multiple transcription factors are known to be involved in the development of choroid plexus epithelial cells from neuroependymal cells (Liddelow, 2015). The cells contribute to the composition of the CSF by e.g., active transport of sodium ions and by secretion of the mitogen amphiregulin among other factors (Falk and Frisen, 2002; Christensen et al., 2013). Conversely, ependymal cells also transport waste products and harmful substances from the CSF into the blood and thus are critical regulators of homeostasis of the central nervous system (Spector et al., 2015). It is therefore not surprising that the CP is associated with a number of diseases including hydrocephalus (Wolburg and Paulus, 2010), neurodegenerative diseases (Alvira-Botero and Carro, 2010), multiple sclerosis (Vercellino et al., 2008) as well as tumors (Tong et al., 2015).

Regarding CP development and function, different signaling pathways have been identified including Sonic hedgehog (Huang et al., 2009; Nielsen and Dymecki, 2010), BMP (Currle et al., 2005) and Notch (Irvin et al., 2004). A number of studies have utilized zebrafish larvae to gain further insights into CP formation and function (Bill et al., 2008; Garcia-Lecea et al., 2008; Henson et al., 2014; van Leeuwen et al., 2018). While mammals possess four ventricles, zebrafish only has two and therefore also two choroid plexus structures, namely in the diencephalon and myelencephalon. Ventricle opening in zebrafish takes place before the first heart beat at 18–22 h post fertilization (hpf) and the ventricles are shaped by sharply bending regions of the neuroepithelium (Lowery and Sive, 2005). The CP primordium, the *tela choroidea* forms at the dorsal midline at 36 hpf by recruitment of cells from the dorsal neuroepithelium and the rhombic lips. Subsequently, cells from the latter are recruited towards the *tela choroidea* to finally form the CP by active coalescence (Garcia-Lecea et al., 2008).

The Wilms tumor suppressor gene *Wt1* encodes a transcription factor with an important role in development and homeostasis of mesoderm-derived tissues like gonads, kidneys, spleen and heart (Kreidberg et al., 1993; Herzer et al., 1999; Moore et al., 1999; Chau et al., 2011; Dong et al., 2015b). More recently, we and others have shown that inactivation of *Wt1* in the mouse spinal cord or the hindbrain leads to impairments of locomotion behaviour and respiration, respectively (Haque et al., 2018; Schnerwitzki et al., 2018; Schnerwitzki et al., 2020). In both cases, *Wt1* is expressed in a specific subset of neurons that are part of corresponding central pattern generator networks. The latter coordinate the generation of rhythmic activity such as locomotion or respiration. While expression of *Wt1* in the central nervous system has been observed earlier (Sharma et al., 1992; Armstrong et al., 1993), the findings mentioned above show that this transcription factor also exerts a physiological function in the mammalian brain.

Zebrafish possess two *wt1* paralogs, namely *wt1a* and *wt1b*. During development as well as in adults both genes are expressed in an overlapping but not identical spatial and temporal pattern (Bollig et al., 2006). While early expression can most prominently be detected in the intermediate mesoderm and the developing glomeruli, expression of *wt1a* and *wt1b* in the adult kidney is confined to podocytes, cells that wrap around capillaries in the glomerulus. Outside of the kidney significant expression of both genes can be observed in the spleen, the heart and the gonads of adult fish. Functionally, *wt1a* and *wt1b* also differ. Morpholino-mediated knockdown of *wt1a* leads to failure of glomerular differentiation and morphogenesis resulting in a rapidly expanding general body edema, while knockdown of *wt1b* is compatible with early glomerular development (Perner et al., 2007). At later timepoints, *wt1b* morphant embryos develop cysts in the region of the glomeruli and tubules and subsequent pericardial edema at 4 days post fertilization. Thus, *wt1a* plays an early role in pronephros development and is essential for the formation of glomerular structures while *wt1b* functions at later stages of nephrogenesis. The fact that *wt1a*, but not *wt1b*, is an essential gene is reflected by death of *wt1a* mutant larvae before day 13, while *wt1b* mutants do not show obvious phenotypic anomalies (Zhang et al., 2018; Sanz-Morejon et al., 2019).

Here, we report three expression domains of *wt1a* in the developing zebrafish CNS, namely in the dorsal hindbrain, the caudal medulla and the spinal cord. In two of those domains, the caudal medulla and the spinal cord, the *wt1a* expressing cells are neurons. However, in the dorsal hindbrain the Wt1+ cells are ependymal cells of the myelencephalic ventricle. We also show that *wt1a* mutant zebrafish larvae show anomalies in development and structure of the myelencephalic choroid plexus region. Moreover, by using a dye accumulation assay we demonstrate that *wt1a* knockout reduces the blood-cerebrospinal fluid (CSF) barrier function of the CP. Thus, Wt1a plays a role in choroid plexus formation in zebrafish and contributes to the development of the CP barrier function.

## Materials and Methods

### Fish Husbandry

Zebrafish were kept in the FLI fish facility at maximal five fish/l under constant conditions: 26–28°C, 400–800 μS, pH 6.5–7.5, 14-hours-light and 10-hours-dark cycle and 10% of water exchange per day. Animals were fed once a day with artemia and twice with dry food. Embryos were raised at 28°C and staged according to (Kimmel et al., 1995). Experiments were conducted with zebrafish larvae younger than 6 days. The following lines were used: wild-type WTABJxTü, *Tg*(*wt1a:eGFP*)^li1Tg^ (Perner et al., 2007), *wt1b* ^*ex2_del5*^ (Sanz-Morejon et al., 2019) and the newly generated lines *wt1a* ^*ex1_del5*^ and *Tg*(*wt1a:EGFP*)*; wt1a* ^*ex1_del5*^ *.*

### Whole-Mount *in situ* Hybridization

Zebrafish embryos were kept in embryo water containing 0.003% n-phenylthiourea (PTU) to prevent melanization. At distinct stages of development, embryos were anesthetized with tricaine, fixed in 4% paraformaldehyde and processed for whole-mount *in situ* hybridization, essentially as described (Hauptmann and Gerster, 1994). The digoxigenin-labeled *wt1a* riboprobe was synthesized as published (Bollig et al., 2006). Following probe detection, embryos were fixed in 4% paraformaldehyde again, taken through a methanol series and equilibrated and oriented for imaging in a clearing solution (1/3 benzyl benzoate, 2/3 benzyl alcohol). Images and image stacks were acquired using a stereo zoom microscope (Axio Zoom. V16, Zeiss) equipped with a 1 x objective (Plan Neofluar 1x/0.25, FWD 56 mm) and a color camera (AxioCam HRc, Zeiss). Extended depth of focus (EDF) images were generated using the ZEN2 blue software (Zeiss).

### Immunofluorescence on Whole Zebrafish Larvae

Zebrafish larvae were treated with PTU as described above, anesthetized and fixed overnight in 4% paraformaldehyde in PBS. After three washing steps in PBS-Tw0.2 (0.2% Tween in PBS) the samples were used either for immunofluorescence against NeuN or Cldn5.

For NeuN staining, the samples were permeabilized by washing them twice with ddH_2_O (4°C), incubating them for 20 min in pre-chilled acetone (−20°C) and washing them three times with ddH_2_O. Afterwards the samples were washed for several times in PBS-Tw0.2 and blocked for 2 h at RT in blocking buffer (10% NGS, 0.8% Triton X 100, and 1% BSA [w/v] in PBS-Tw0.2). Samples were incubated for 3 days at 4°C with *a*-NeuN (Abcam plc., United Kingdom: ab177487, rabbit), diluted 1:100 in incubation buffer (1% NGS, 0.8% Triton X 100, and 1% BSA [w/v] in PBS-Tw0.2). After washing for three times for 1 h in PBS-TN (10% NGS; 1% Triton X 100 in PBS), twice for 10 min in PBS-Tx1 (1% Triton X-100 in PBS), and again two times for 1 h in PBS-TN, the samples were incubated for 1 day at RT with the secondary antibody *a*-rabbit Alexa Fluor^®^ 546 (Thermo Fisher Scientific Inc., A-11071, goat), diluted 1:300 in incubation buffer. Finally, the samples were washed three times with PBS-TN (for 1h; each) and several times with PBS-Tw0.2.

For staining against Cldn5, the samples were washed once briefly with PBS-Tx1 and three times for 10 min with PBS-Tx0.1 (0.1% Triton X 100 in PBS). They were permeabilized by incubation in 2.5% Trypsin solution in PBS (Biochrom Trypsin 2.5% w/o Ca, Mg) for 10 min and then washed three times in PBSTx0.1. The larvae were treated for 3 h in blocking buffer (10% NGS in PBSTx1) and incubated for 2.5 days at 4°C with *a*-Cldn5 (Thermo Fisher Scientific Inc., United States: RH235294, mouse), diluted 1:100 in incubation buffer (1% NGS and 1% BSA in PBSTx1). After a brief washing step in PBSTx1, the larvae were washed three times in PBSTx0.1 and blocked for 1 h in blocking buffer. The samples were then incubated at 4°C with the secondary antibody *a*-mouse Alexa Fluor^®^ 546 (Thermo Fisher Scientific Inc., A-11018, goat) diluted 1:400 in incubation buffer for ∼24 h. Finally, the larvae were washed several times in PBSTx1 and stored in PBSTx0.1 until imaging.

### Immunofluorescence on Tissue Sections

For cryosections, zebrafish larvae were either fixed before embedding in 4% paraformaldehyde in PBS as described above (prefixation) or directly embedded in cryosection medium (postfixation) under anesthesia (0.16 mg/ml tricaine in Danieau´s solution). Then, the larvae were dehydrated with 20% sucrose (in 50% NEG-50™/PBS medium) for 15 min, embedded in a mold filled with NEG-50™ cryosection medium, and frozen. Subsequently, transverse sections of 20 µm were made. The sections without prior fixation were post-fixed for 10 min and washed four times for 10 min at RT in PBS-Tw0.2. Hereafter, only prefixed sections were permeabilized by washing with permeabilization solution (0.1% Tween, 0.3% Triton X 100 in PBS) for some seconds. Immediately after, the sections were washed with PBS-Tw0.2 (twice for 10 min). After this step, pre- and post-fixed samples were treated the same. After blocking with blocking buffer (2% BSA and 10% NGS in PBSTw0.2) for 1 h at RT, the samples were incubated over night at 4°C with the diluted primary antibody in blocking buffer: *a*-Elavl3+4 1:200 (GeneTex, Inc.: GTX128365, rabbit); *a*-NeuN 1:100 (Abcam plc., United Kingdom: ab177487, rabbit); *a*-Sox2 1:200 (Abcam plc., United Kingdom: ab97959, rabbit); *a*-Cldn5 1:100 (Thermo Fisher Scientific Inc., United States: RH235294, mouse); *a*-GFP 1:200 (Thermo Fisher Scientific Inc., United States: A-11122, rabbit); *a*-Acetylated Tubulin 1:500 (Sigma-Aldrich Chemie GmbH Munich, Germany: T 6793, mouse). The slides were then washed several times in PBS-Tw0.2 at RT. Depending on the host species of the primary antibody, one of the following corresponding secondary antibodies was chosen and diluted in blocking buffer: *a*-rabbit Alexa Fluor^®^ 546 1:500 (Thermo Fisher Scientific Inc., A-11071, goat); *a*-rabbit Cy5-AffiniPure 1:500 (Dianova, Germany, 711-175-152, donkey); *a*-mouse Cy5-AffiniPure 1:500 (Dianova, Germany, 115-175-146, goat). Bisbenzimide Hoechst 33,258 was used for nuclear staining. After 1 h incubation at RT, the slides were washed several times in PBS-Tw0.2. Finally, the slides were mounted with 70 μl ProLong^®^ Gold or ProLong^®^ Diamond antifade reagent (Thermo Fisher Scientific Inc.) and incubated overnight at 4 °C.

### Fluorescence Imaging

Images and image stacks of sections were acquired using an Axio Imager. Z1 equipped with an ApoTome.2 slider for optical sectioning (Zeiss, Germany). For image processing the ZEN2 blue software (Zeiss, Germany) was used and either single images or extended depth of focus projection of z-stacks were displayed. Whole mount larvae were either imaged as described (α-NeuN staining) or with a light sheet microscope (α-Cldn5 staining, morpholino experiments). For light sheet microscopy, a Lightsheet Z1 (Zeiss) enabled for dual side illumination and equipped with a 20x detection objective (W Plan-Apochromat, numerical aperture = 1.0) and a sCMOS pco. edge 4.2 camera was employed. Image processing consisting of dual side fusion, brightness/contrast adjustment and unsharp masking was performed by using the Zen 3.1 (blue edition) software (Zeiss, Germany). For three-dimensional reconstruction the 3Dxl rendering module (powered by arivis, Germany) was used.

### Morpholino-Mediated Knockdown Experiments

For knockdown experiments, morpholino antisense oligonucleotides (Gene Tools, LLC, Philomath, United States) were diluted in water to a working concentration of 1 mM with 0,05% phenol red (Sigma) as a tracer. Microinjections of 0.2 pmol morpholino into the yolk of 1-to 2-cell embryos were performed using a manual micromanipulator (type M1, Saur, Germany). The injection amount was calculated by employing a graticule (Pyser–SGI, Edenbridge, United Kingdom). The *wt1a* morpholino was designed to target the first splice donor site causing an intron inclusion (AAA​GTA​GTT​CCT​CAC​CTT​GAT​TCC​T). As a control, a standard MO that targets a human *beta-globin* gene intron mutation was injected (CTC​TTA​CCT​CAG​TTA​CAA​TTT ATA).

### Generation of wt1a Mutant Zebrafish and Genotyping of wt1a and wt1b Mutants

The *wt1a* mutant line was generated by CRISPR/Cas9-mediated genomic engineering. Fertilized wild-type AB zebrafish oocytes were injected with a solution containing the single guide sgRNA (15 ng/μl, UCA​UCA​AGC​AGG​AGC​CCA​GUU​GG), *cas9* mRNA (150 ng/μl) and phenol red. From several *wt1a* mutant alleles that were identified, a line was generated (following *WTABJxTü* outcross) harboring a five base pair deletion within exon 1 of *wt1a* and named *wt1a* ^*ex1_del5*^ (Figure 3A). The specific allele is predicted to cause a premature stop codon (Figure 3A). The *Tg(wt1a:EGFP); wt1a* ^*ex1_del5*^ line was generated by crossing the transgenic *Tg(wt1a:EGFP)* line (Bollig et al., 2009) with the newly generated *wt1a* mutant line. The resulting animals were subsequently intercrossed. After microscopic screening for EGFP fluorescence genotyping was performed as described below.

Genotyping of *wt1a* mutant zebrafish was performed by the HRMA (High Resolution Melt Analysis) method. Zebrafish larvae were anesthetized and tissue was removed by cutting the caudal fin caudally of the cardinal vein. The biopsy material was transferred into 15 μl 50 mM sodium hydroxide into a PCR tube, heated to 98°C for 2 min and subsequently neutralized with 1.6 μl 1 M Tris-HCl (pH 8.0). After centrifugation, the lysate was used for HRMA analysis. To this end, 1 μl of the lysate was mixed with 9 μl master mix containing 5 µl of Precision Melt Supermix (BioRad Laboratories), 0.5 μl each of 10 μM primers *wt1a_HRMA_for (GGT TCT GAT GTT CGT GAC)* and *wt1a_HRMA_rev (TGG AAG AGT ACA GTT ACC GTT TC)*. The amplicon comprises 81 bp in wild type and 76 bp in mutant animals. PCR was carried out in a 384-well format using the CFX384 Touch real-time PCR Detection System (Bio-Rad Laboratories) for 42 cycles, followed by high-resolution melting analysis. Genotypes were determined using the Precision Melt Analysis™ Software (Bio-Rad).

For genotyping of *wt1b* mutants, a Kompetitive Allele Specific PCR (KASP) assay was developed together with LGC Bioresearch Technologies. For allelic discrimination the primers ACA​GTA​GCA​GTC​GAC​GGA​ATT​CCC​AGT​TAC​GGT​CAC​ACA​CCT​ACA​CAA​CAC​T-CTC​CT (wild type, FAM-labelled) and ACA​GTA​GCA​GTC​GAC​GGA​ATT​CCC​AGG​TCA​CAC-ACC​TAC​ACA​ACA​CTC​TCC​TCC​G (mutant, HEX-labelled) were used. KASP assays were performed in 96 well plates. Genomic DNA was cleaned with the QIAamp DNA Micro Kit (Qiagen). For each reaction, 5 μl of cleaned DNA, 5 μl of KASP Master Mix and 0.14 μl of the KASP primer mix were used. The KASP assay was performed on a CFX96TM Real-Time System (Biorad). Data analysis was performed with the software Bio-Rad CFX Manager.

### Quantitative Real-Time PCR

Total RNA was isolated from pools of three five dpf zebrafish larvae using TRIzol (Invitrogen). Subsequently, 500 ng RNA was reverse transcribed with the iScript cDNA Synthesis Kit (Bio-Rad). All qPCR assays were performed in triplicates using 15 ng of cDNA per reaction. The qPCR program (95°C for 15s, 55°C for 20s, 60°C for 40s, 39 cycles) was run and recorded via the CFX384 Touch real-time PCR detection system (Bio-Rad) using SYBR^®^ GreenER™ qPCR SuperMix for iCycler (Invitrogen). *Ef1a* was used as a reference gene. The data were analyzed using the efficiency-corrected relative quantification model (Pfaffl, 2001). Primers for RT-PCR were *podocinF* GCT​CAG​GAG​ATT​CAG​GTC​ACT, *podocinR* TGC​AGC​TCT​GGA​GGA​AGA​TT, *ef1aF* AAG​AGA​ACC​ATC​GAG​AAG​TTC​GA and *ef1aR* ACC​CAG​GCG​TAC​TTG​AAG​GA.

### Fluorescent Tracer Injection/Detection and Dye Accumulation Assay

Injection needles were pulled using a Micropipette Puller P-97 (Sutter Instrument) and glass capillaries with filament. Needles were filled with a solution of 7 mg/ml each of tetramethylrhodamine- and fluorescein-labeled dextrans of molecular weight 10 and 40 kDa, respectively (both Thermo Fisher Scientific). At 5 days post fertilization (dpf) zebrafish larvae were anesthetized using 0.16 mg/ml tricaine, embedded in *µ*-Dishes (ibidi GmbH, Germany) containing 1% low melting agarose with 0.16 mg/ml tricaine and injected into the cardinal vein with approximately 1–2 nL using a Micromanipulator and a Pneumatic Pico Pump PV280 (both H. Saur Laborbedarf, Germany). In each case one wild type and one mutant larva were embedded together in one *µ*-Dish for injection and imaging. Imaging was performed at 30, 35, 40, 45 and 50 min after injection using the stereo zoom microscope Axio Zoom V16 (Zeiss) equipped with a 1 x objective (Plan Neofluar 1x/0.25, FWD 56 mm) and a mono camera (AxioCam MRm, Zeiss), a motorized stage and modules for tiling, time series and autofocus correction. The ZEN2 blue software was used to manage image acquisition. Quantification of fluorescence intensities was performed using Fiji (ImageJ). To compensate for different injection efficiencies the mean fluorescence intensity of the respective ventricle was normalized to the fluorescence intensity of the pupils of the same larva at the same time point and is given as mean intensity ratio [ventricle/pupil]. To assess the significance of effects for the different parameters, a two-way ANOVA was performed using the SPSS software. Parameters considered were time, molecular weight (kDa) and genotype as well as the interaction term between time and genotype. Significance was determined as * = *p* < 0.05, ** = *p* < 0.01, *** = *p* < 0.001.

## Results

### *Wt1a* Is Expressed in Three Domains in the Zebrafish CNS

**FIGURE 1:**
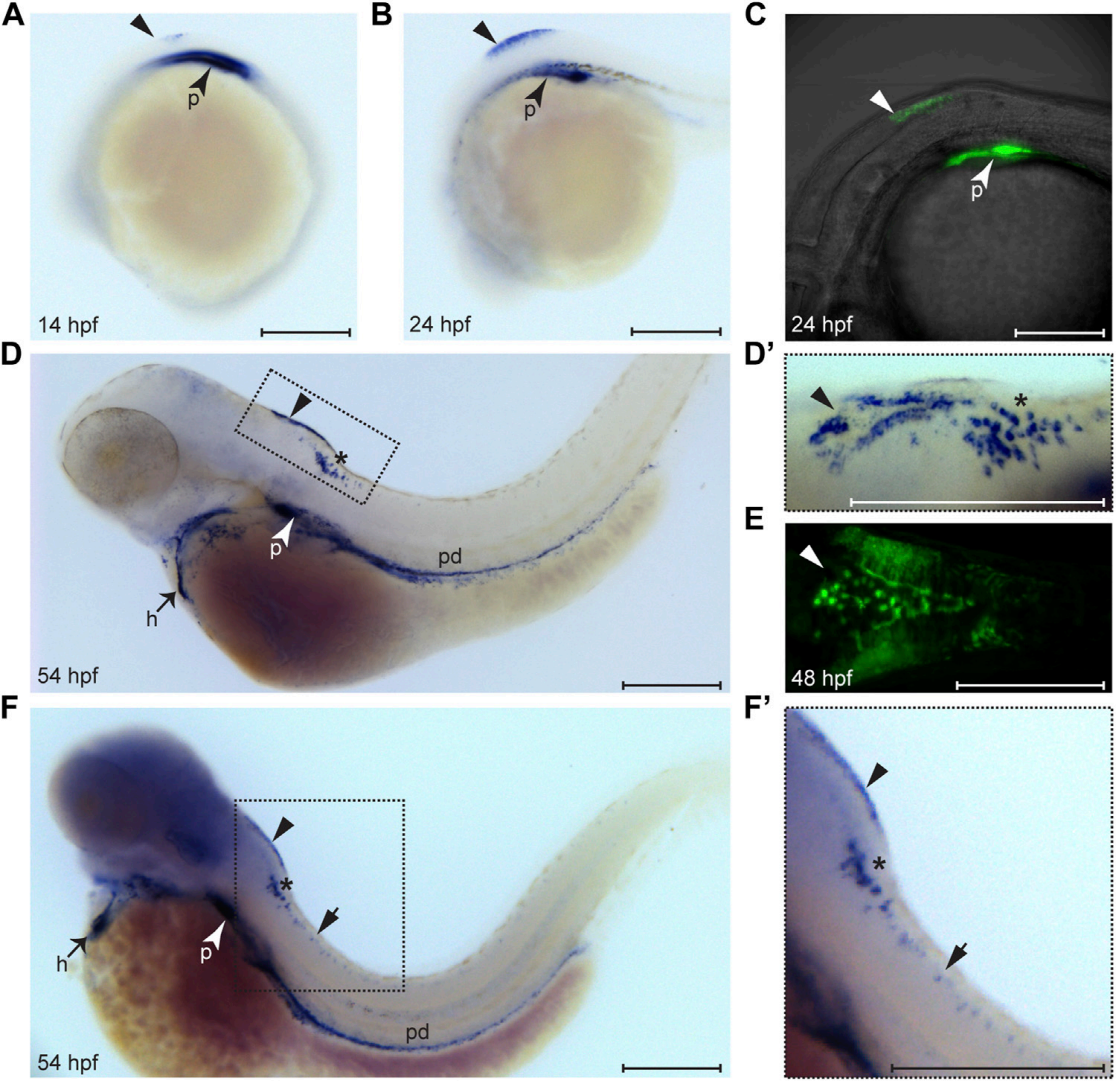
Expression pattern of *wt1a* during embryonic and larval development. *Wt1a* expression was analyzed by whole mount *in situ* hybridization **(A, B, D, D′, F, F′)** and via examination of the EGFP signal in animals carrying the *wt1a:EGFP* transgene (C, E). **(A)** In addition to the known and prominent expression in the developing pronephros (arrowhead), weak expression of *wt1a* is present in the presumptive dorsal hindbrain at 14 hpf (triangle). **(B)** The dorsal hindbrain domain appeared more prominent at 24 hpf. **(C)** Overlay of transmission and fluorescent images of a *Tg*(*wt1a:EGFP*) embryo reveals that the domain described in B is located at the most posterior region of the hindbrain. **(D)** At 54 hpf *wt1a* expression is detected in the pronephros, in the heart, along the pronephric duct and in two domains of the hindbrain. In addition to the above mentioned very dorsally located domain (arrowhead) the *wt1a* transcript is also present in a more ventral region (asterisk). **(D′)** A closer, dorsolateral view on both hindbrain domains illustrates that the dorsal domain (arrowhead) has a triangular shape with a compact structure in the middle. **(E)** The EGFP signal arising from a 2 day old embryo of the *wt1a* transgenic line closely recapitulates the v-shaped expression described in D’. **(F-F′)** Upon extended staining of a 54 hpf larva *wt1a* expressing cells are also detectable in the spinal cord (short arrow). A-D, F and F′ are lateral views, D′ is a dorsolateral and E a ventral view. All images are with rostral to the left. h: heart; p: pronephros; pd: pronephric duct. Scale bars: 200 μm.

We performed a detailed expression analysis of *wt1a* in zebrafish larvae using *in situ* hybridization and discovered three prominent sites of expression within the central nervous system, namely in the dorsal hindbrain, the caudal medulla and the spinal cord. *Wt1a* expression in the brain becomes detectable as early as 14 h post fertilization (hpf) with a more prominent signal at 24 hpf (Figures 1A,B). Interestingly, this expression is also reflected in an EGFP signal of the *Tg(wt1a:EGFP)* line (Figure 1C) suggesting that this line faithfully reflects endogenous *wt1a* activity (Bollig et al., 2009). At 54 hpf, *wt1a* expression in the brain can be seen in two domains: in a triangular shape in the dorsal hindbrain and in a scattered pattern in the caudal medulla (Figure 1D and D′). Particularly the signal in the dorsal hindbrain can also be detected in the *Tg(wt1a:EGFP)* line (Figure 1E). Upon extended staining, *wt1a* expression can also be seen in single cells along the spinal cord (Figure 1F,F’), a domain for which *Wt1* expression in the mouse has recently been reported (Haque et al., 2018; Schnerwitzki et al., 2018). To assess whether we can also detect Wt1a protein in the domains mentioned above, we performed immunohistochemistry using two different *a*-Wt1 antibodies that had been generated against the mammalian Wt1 protein. Both antibodies detected the same cells within the three *wt1a* expressing domains in 3-days old zebrafish larvae (Supplementary Figure 1). Thus, both antibodies could be used for further characterization.

### Wt1a + Cells in the Hindbrain Are Ependymal Cells of the Choroid Plexus

**FIGURE 2:**
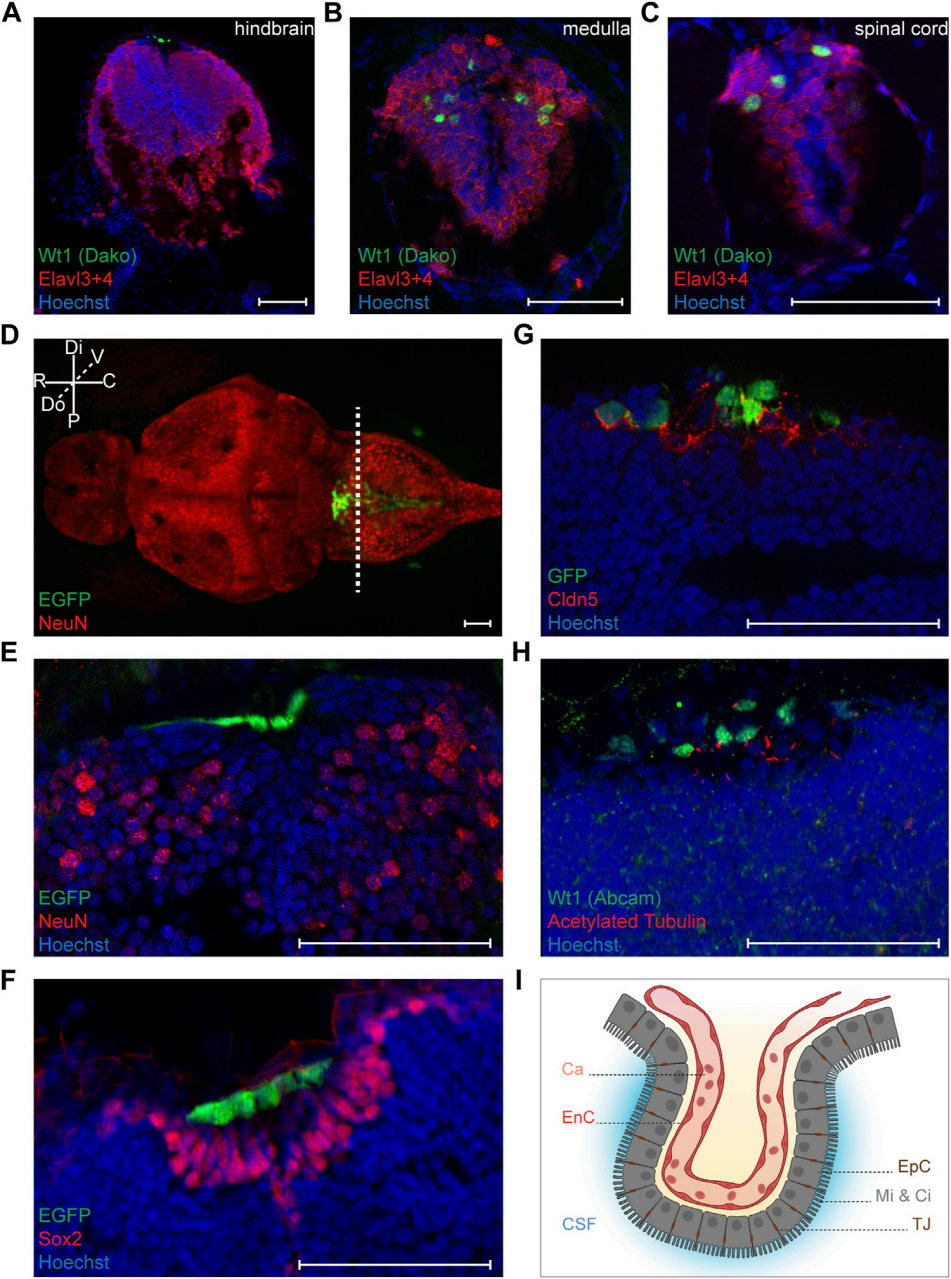
Characterization of Wt1+ cells of the zebrafish larval CNS. **(A-C)** Transverse sections through the hindbrain, the caudal medulla and spinal cord of 4.25 days old *Tg(wt1a:EGFP)* zebrafish larvae were stained with antibodies against Wt1 (Dako) and Elavl3+4. The images show optical sections of one focus plane. Orientation: dorsal: up. Scale bar: 50 µm. **(D)** Whole zebrafish larvae (4.5 dpf) of the *Tg(wt1a:EGFP)* line were used for detection of the EGFP signal of Wt1 positive cells and IHC against NeuN. The image is displayed as extended depth of focus projection. The fluorescence signal for EGFP does not overlap with NeuN staining. The dotted line indicates the cutting position of the following transverse sections. Orientation: rostral: left; dorsal view. Abbreviations: distal (Di); proximal (P); rostral (R); caudal (C); dorsal (Do); ventral (V) Scale bar: 50 µm. **(E)** Transverse section through the hindbrain of 4.25 days old *Tg(wt1a:EGFP)* zebrafish larvae were used for detection of the EGFP signal of Wt1 positive cells and IHC against NeuN. **(F)** Transverse sections through the hindbrain of 4.25 days old *Tg(wt1a:EGFP)* zebrafish larvae were used for detection of the EGFP signal of Wt1 positive cells and IHC against Sox2. **(G)** Transverse sections through the hindbrain of 4.25 days old *Tg(wt1a:EGFP)* zebrafish larvae were used for IHC against GFP and Cldn5. **(H)** Transverse sections through the hindbrain of 4.25 days old *Tg(wt1a:EGFP)* zebrafish larvae were used for IHC against Wt1 (Abcam) and acetylated Tubulin. (E–H) The images show optical sections of one focus plane. Orientation: dorsal: up. Scale bar: 50 µm. **(I)** Schematic illustration of the ependymal cells of the fourth brain ventricle, which are connected via tight junctions and show microvilli and cilia at their apical surface. The image was created with BioRender.com. Abbreviations: capillary (Ca); endothelial cell (EnC); cerebrospinal fluid (CSF); ependymal cells (EpC); microvilli (Mi); Cilia (Ci); tight junction (TJ).

We next wanted to characterize the *wt1a* expressing cells in the zebrafish CNS more closely. For this we used the *Tg(wt1a:EGFP)* line and, dependent on antibody compatibility, performed staining against Wt1 or GFP. Alternatively, we detected EGFP fluorescence. Staining of sections through the hindbrain revealed that the Wt1a + cells of this domain did not show an overlap with the neuronal marker Elavl3+4 (Figure 2A). In contrast, Wt1+ cells in both the medulla oblongata and the spinal cord were also detected with the antibody against Elavl3+4 (Figures 2B,C). This shows that the *wt1a* expressing cells in the caudal medulla and the spinal cord, but not in the hindbrain are neurons. To confirm this observation, we employed an antibody against another neuronal marker, NeuN. Again, the respective signal showed no overlap with the EGFP signal of the transgenic line (Figure 2D). The EGFP positive cells appeared more dorsal than the NeuN positive cells (Figure 2E) and did also not stain positive for Sox2, a marker for neuronal stem and progenitor cells (Figure 2F).

After having shown that the Wt1a + cells of the dorsal hindbrain do not belong to the neuronal lineage, we hypothesized that they might be part of the choroid plexus. The cells of the CP possess tight junctions, which contain the Claudin 5 (Cldn5) protein (Henson et al., 2014). Staining of 4.25 days old zebrafish larvae with an antibody against Cldn5 revealed that the EGFP positive cells in the dorsal hindbrain of *Tg(wt1a:EGFP)* larvae were positive for Cldn5 (Figure 2G). This suggests that *wt1a* expressing cells are part of the CP of the myelencephalic ventricle in the zebrafish brain. To discriminate between ependymal and endothelial cells we made use of the fact that ependymal cells possess microvilli and cilia on their apical surface, while endothelial cells lack these structures (Mirzadeh et al., 2017; Shah et al., 2018). When we employed an antibody against acetylated tubulin that detects cilia and microvilli, we found that most of the Wt1 positive cells possessed cilia on their surface (Figure 2H). Hence, it can be assumed that Wt1 positive cells of the zebrafish myelencephalic choroid plexus (mCP) are ependymal cells (Figure 2I).

### *Wt1a* Mutant Zebrafish Do Not Survive Beyond Day 10

**FIGURE 3:**
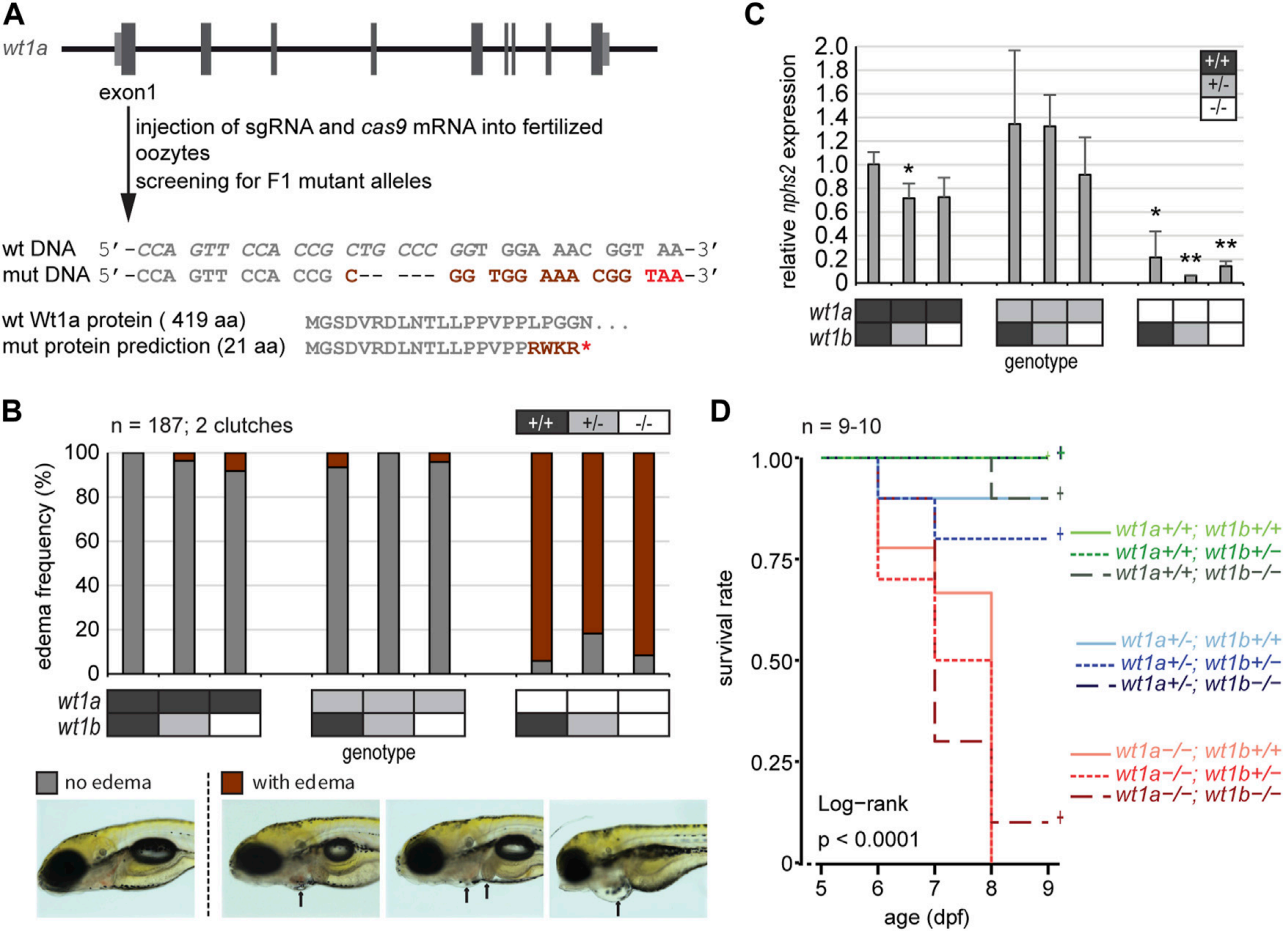
Inactivation of *wt1a* results in edema formation and early lethality. **(A)** Schematic drawing of the *wt1a* gene locus and mutant generation. With the help of CRISPR/Cas, a fish line was established that lacks five nucleotides in exon 1 of the *wt1a* gene (*wt1a* ^*ex1_del5*^). The sgRNA target sequence is italicized in the wild type DNA sequence. The 5 nt deletion is predicted to result in a frame shift (dark red) and a premature termination codon (red) that leads to a truncation of the protein. Abbreviations: wt (wild type); mut (mutant). **(B)** Offspring of a double heterozygous incross (*wt1a* ^*+/*-^;*wt1b* ^*+/*-^) were screened for the formation of edema at five dpf. The majority of *wt1a* ^−/−^ larvae have body edemas (arrows), independent of the *wt1b* ^*ex2_del5*^ background. Example pictures for no edema (grey), and body edemas (dark red) are shown; n = 187. **(C)** qRT-PCR analysis of offspring of a double heterozygous incross (*wt1a* ^*+/*-^;*wt1b* ^*+/*-^) indicates a strong reduction of *nphs2* expression in *wt1a* ^−/−^ larvae. n = 3 (three to five larvae each). Error bars represent standard error. One-way ANOVA was calculated as *p* = 3.98*10^–05^. *p*-values are calculated with a *t*-test by pairwise comparison to wild type (*wt1a* ^+*/*+^; *wt1b* ^+*/*+^). **(D)** Survival of offspring of a double heterozygous incross (*wt1a* ^*+/*-^;*wt1b* ^*+/*-^) from five to nine dpf shows an early onset of lethality of homozygous *wt1a* ^*−/−*^ larvae, independent of the *wt1b* ^*ex2_del5*^ background. Survival is shown as a Kaplan-Meier curve. n = 9–10 larvae per genotype.

In order to assess whether Wt1a also contributes to the structure and function of the mCP, we generated a mutant zebrafish line for *wt1a*. We employed CRISPR/Cas9 and a single guide RNA targeting exon 1 of *wt1a*. From the resulting alleles, a five base pair deletion within exon 1 of *wt1a* was selected and propagated in a heterozygous state (*wt1a* ^+/-^). The mutation is predicted to result in a premature stop codon after 21 amino acids (Figure 3A). For the characterization of this line and to consider potential redundancies of the two *wt1* paralogous genes we crossed the *wt1a* ^+/-^ line to the *wt1b* ^+/-^ line that we had generated earlier (Sanz-Morejon et al., 2019). Genotyping of offspring from an in-cross of double heterozygous parents showed that at five dpf all genotypes occurred according to Mendelian ratios. However, at this time point, a very large fraction of *wt1a* ^−/-^ animals had developed visible pericardial edema (Figure 3B). This was not the case in heterozygous *wt1a* mutants and was also independent of the *wt1b* genotype. Edema formation was paralleled by a significant decrease in expression of *nphs2* (*podocin*) (Figure 3C), a marker of the kidney glomerulus and Wt1a/b target gene (Dong et al., 2015b). At 9 dpf almost all *wt1a* mutants had developed severe edema and had to be euthanized, while both heterozygous *wt1a* as well as *wt1b* mutant larvae were indistinguishable from wild types (Figure 3D). This analysis demonstrates that we had generated a true loss-of-function *wt1a* allele with significant phenotypic consequences, which were unaffected by the status of the paralogous *wt1b* allele.

### *Wt1a* Morphants and Mutants Show Anomalies in Choroid Plexus Development

**FIGURE 4:**
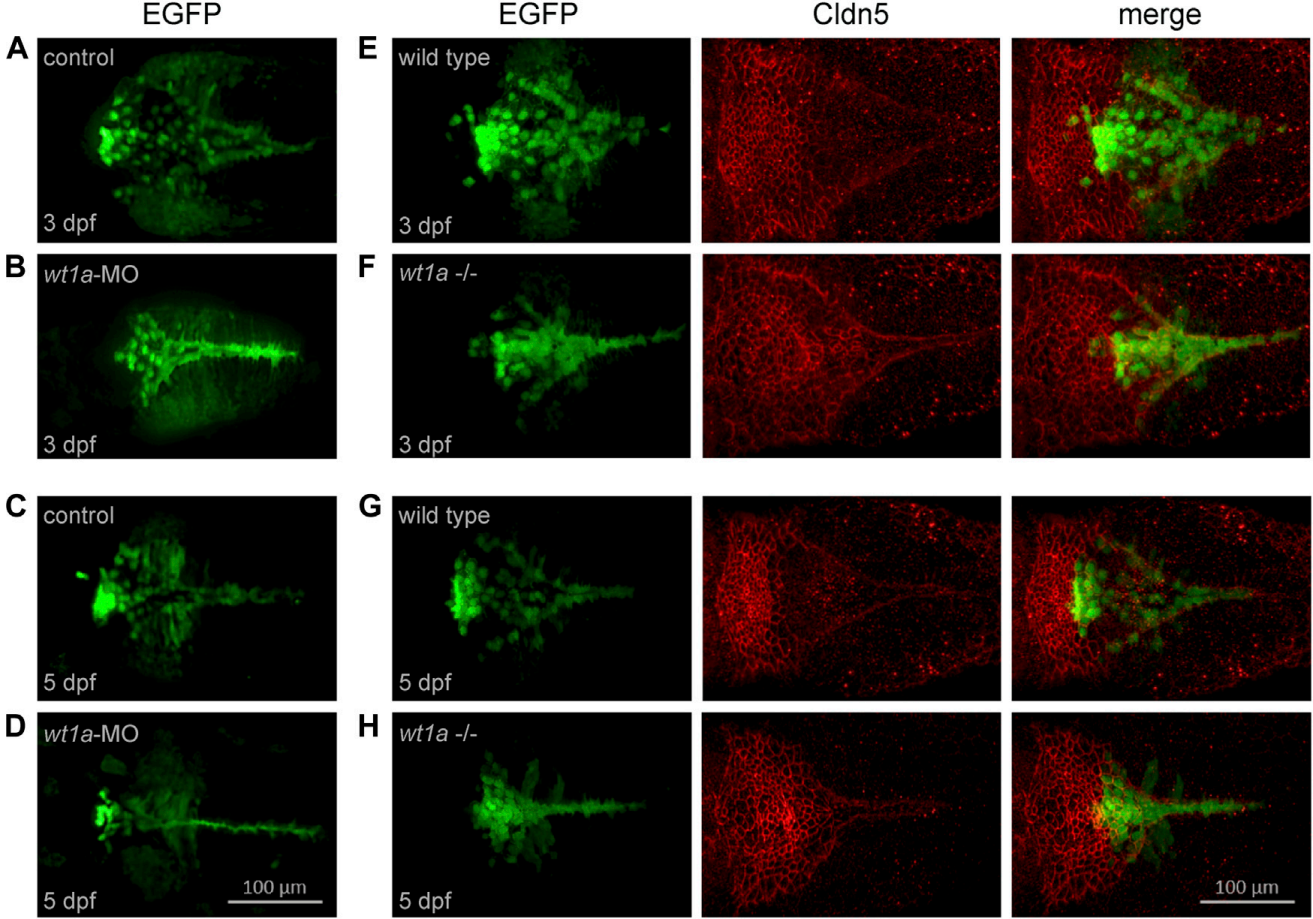
*Wt1a* morphants and mutants show anomalies in choroid plexus development. For the analysis of morphants the *Tg(wt1a:EGFP)* line and for mutants the *Tg(wt1a:EGFP); wt1a* ^*ex1_del5*^ line has been used. **(A–D)** Wt1+ positive cells, visualized by EGFP fluorescence, in zebrafish larvae injected with control morpholino (A,C) or *wt1a* morpholino (B,D) at three dpf (A,C) and five dpf (B,D). **(E–H)** Wt1+ positive cells **(*left*)**, visualized by EGFP fluorescence in wildtype (E,G) or homozygous *wt1a* mutant larvae (F,H) of the *Tg(wt1a:EGFP)* line were stained with *a*-Cldn5 antibody **(*middle*)** at three dpf (E,F) and five dpf (G,H). All panels represent 3D reconstructions of image stacks (dorsal views with rostral to the **left**).

In a next step we wanted to study the consequences of *wt1a* inactivation for CP development and structure. For this we used two approaches, namely morpholino-mediated knockdown and CRISPR/Cas9-mediated genetic inactivation. For both experimental strategies we used the background of the *Tg(wt1a:EGFP)* line. In control zebrafish larvae the Wt1+ cells in the hindbrain present themselves in form of a zipper-like structure that is open at its rostral and closed at its caudal end (Figures 4A,C). In addition, a cluster of strongly positive and aggregated cells can be found at the rostral end, slightly anterior to the base of the triangular field. Also, two areas of weakly positive cells are located on both sides of the triangle. This structure is visible at both three and five dpf, however, at the later timepoint the structure is more compact and strongly positive cells appear to be increasingly aggregated at the rostral cluster. Upon morpholino-mediated knockdown, the area of Wt1+ cells becomes significantly more narrow with thinning of the triangle to a line. Furthermore, the rostral cluster is either lacking (Figure 4B) or appears less prominent (Figure 4D).

In the *wt1a* mutant very similar changes with regard to the location of Wt1+ cells could be seen. Again, the area of Wt1+ cells was more compact than in wild type controls (Figures 4E–H). In order to analyze the impact of *wt1a* inactivation on overall mCP formation, we performed staining of wild type and *wt1a* mutant larvae with an antibody against the CP marker Cldn5. In wild type larvae the rostral part of the mCP appears as a tight network of cells, while in the more caudal part, the Cldn5 positive cells are arranged in a triangular-shaped and loose structure (Figures 4E,G). The morphology of the mCP was less densely packed in *wt1a* mutants and appeared to be more planar, whereas the caudal triangular shape of the Cldn5 signal was less extended in caudal direction and more narrow as well as more intense (Figures 4F,H). Additionally, the transition of the rostral cluster to the caudal triangle was less demarcated in mutant larvae. Thus, inactivation of *wt1a* changes the morphology of the zebrafish mCP.

### *Wt1a* Inactivation Reduces the Blood-CSF Barrier Function

**FIGURE 5:**
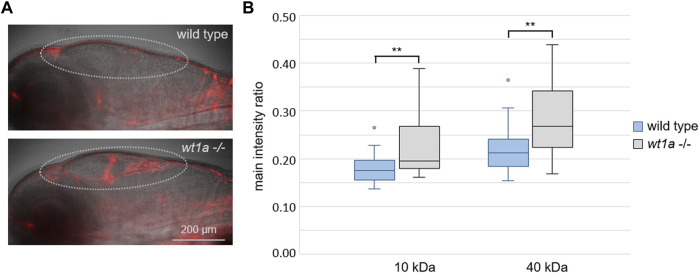
*Wt1a* inactivation reduces the blood-CSF barrier function. Fluorescently labeled dextrans were injected into the cardinal vein of 5 days old larvae of the *wt1a* ^*ex1_del5*^ line and leakage into the ventricles was analyzed. **(A)** Representative overlay images of transmission and rhodamin-dextran (10 KDa) fluorescence recorded by light sheet microscopy of larvae that were fixed 4 h after injection. The dotted line marks the ventricular system. **(B)** Ratios of fluorescence intensities of rhodamin-dextran (10 KDa) and fluorescein-dextran (40 kDa) in the ventriclular region relative to the respective fluorescence in the pupil were estimated *in vivo* 40 min post injections. n = 24 for wild types and 22 for mutants; *p*-values according to ANOVA: 0.003 and 0.002 (**) for 10 and 40 kDa, respectively.

Since mCP morphology was altered in *wt1a* mutant larvae, we wanted to assess whether inactivation of *wt1a* also affects the function of the blood-CSF barrier of the mCP. To this end, we injected rhodamin-labeled dextran (10 kDa) into the larval blood stream, fixed the larvae 4 h post injection and visualized the dextran accumulation in the ventricles by light sheet microscopy. While hardly any red fluorescence signal could be observed in wild type larvae, a considerable amount of the fluorescent tracer escaped from the blood into the ventricular system of *wt1a* mutants (Figure 5A). This suggests that *wt1a* depleted zebrafish larvae fail to build up a tight blood-CSF-barrier in the myelencephalic ventricle. To make sure that *wt1a* mutant larvae do not show a general developmental delay, we measured and compared pupil areas of wild type and mutant larvae. The pupil areas did not differ between the genotypes (Supplementary Figure 2A). When we determined ventricle areas, we found that mutant larvae had a significantly increased ventricle size compared to wild type larvae (10.8 vs. 9 μm^2^, Suppementary Figure 2B). These enlarged brain ventricles point to a hydrocephalus-like phenotype in *wt1a* mutants.

To quantify the accumulation of fluorescent tracers, we performed an *in vivo* dye accumulation assay (adapted from Henson et al., 2014). For this we injected fluorescent dextrans of different colours and sizes (rhodamin and fluorescein, 10 and 40 kDa, respectively) into 5 days old larvae and imaged them at 30, 35, 40, 45 and 50 min post injection. Statistical analyses of the image based quantification of the normalized mean fluorescence intensities in the ventricular regions revealed significant differences between wild type and *wt1a* mutant larvae for both fluorescent dextrans at all time points measured. The exemplary data at 40 min post injection show the significantly higher fluorescence in the ventricles of mutant larvae in comparison to wild type controls (Figure 5B). The increased leakage of both tracers from the blood into the ventricular system suggests an impaired blood-CSF-barrier function in the brain of *wt1a* depleted zebrafish larvae.

## Discussion

In this work we show that one of the two paralogs of the Wilms tumor transcription factor Wt1, namely Wt1a, has a role in formation and function of the choroid plexus of the myelencephalic ventricle in zebrafish. This adds to a growing list of reports that demonstrate that in addition to its canonical role in the differentiation and homeostasis of mesoderm-derived tissues like kidney and gonads, Wt1 also functions in the vertebrate central nervous system and thus in cells of ectodermal origin. This has meanwhile been shown for the spinal cord as well as for the hindbrain of mice (Haque et al., 2018; Schnerwitzki et al., 2018; Schnerwitzki et al., 2020). Our data demonstrate that also in zebrafish *wt1a* is expressed in spinal cord neurons. Whether those neurons fulfil a similar function in zebrafish like in mouse, namely the coordination of locomotion remains to be determined. A recent report, however, suggest this to be the case (Del Pozo et al., 2020).

In case of the choroid plexus, *wt1a* is expressed in non-neuronal ependymal cells. Ependymal cells are derived from embryonic neuroepithelial cells. A very recent report suggests that a common progenitor pool for both epithelial and neuronal cells exists in the CP (Dani et al., 2021). To analyze the role of *wt1a* in mCP formation and function we have generated a respective loss-of-function allele in zebrafish. Homozygous *wt1a* mutant larvae developed significant edema, similarly to the respective morphants. The latter develop pericardial edema at around four dpf and subsequently suffer from rapidly expanding general body edema resulting in death of the larvae (Perner et al., 2007). Also, *wt1a* mutants died at around 10 days after fertilization. The severity of the phenotype as well as survival was not influenced by the state of the paralogous gene *wt1b*, suggesting that there is no compensation. Our findings thus confirm an earlier report on a *wt1a* mutant zebrafish allele, which reported lethality of homozygous *wt1* mutants before 13 dpf (Zhang et al., 2018). The alternative gain-of-function approach is hampered by the dramatic consequences of *wt1a* overexpression. The injection of even very small amounts of *wt1a* mRNA resulted in strong defects including formation of edema as well as anomalies in eye, pronephros and forebrain development (Perner et al., 2007; Schnerwitzki et al., 2014).

Regarding the structure of the mCP we observed significant changes in *wt1a* mutants. The area of the Wt1+ cells in the mutants appeared significantly more compact and narrower than in wild type larvae. This was not caused by a developmental delay as assessed by pupil size, which did not differ between the genotypes. The changes regarding the localization of the Wt1+ cell could also be observed in *wt1a* morphants. When we stained for the tight junction marker Cldn5 we could observe that the rostral part of the CP was less structured and the caudal triangular extension was less extended in mutant larvae. A comparison of the mCP morphological changes in *wt1a* morphants and mutants with normal mCP development (Garcia-Lecea et al., 2008; van Leeuwen et al., 2018) leads us to assume that *wt1a* depletion interferes with early morphological processes. Given that the mCP in zebrafish forms through cell migration rather than cell division (van Leeuwen et al., 2018) it is tempting to speculate that Wt1a plays a role in cell specification associated with migratory behavior. Functionally, we could also show that *wt1a* mutant larvae display a reduced barrier function for substances from the blood entering the cerebrospinal fluid. This was demonstrated by the accumulation of dye-labelled dextrans of 10 and 40 kDa that accumulated in the ventricles of mutant larvae. The decreased barrier function of the mCP in *wt1a* mutants is also demonstrated by an increased ventricle size, resembling a hydrocephalus-like phenotype.

Regarding the possible function of Wt1 in the choroid plexus, some intriguing parallels between the kidney and the CP should be mentioned. While the kidney constitutes a barrier between the blood and the urine, the CP fulfills this function between the blood and the cerebrospinal fluid. Remarkably, it has been shown in rats that the overlap in gene expression profiles between the CP and the kidney is higher than between the CP and other brain regions, namely the cortex or hippocampus (Sathyanesan et al., 2012). In the developing as well as in the mature kidney *Wt1* expression is most prominent in the podocytes (Mundlos et al., 1993). Podocytes surround blood capillaries with interdigitating foot processes and are an important component of the kidney filtration apparatus (Pavenstadt et al., 2003). Central for the CP filter are the ependymal cells, which we report here also express a *wt1* paralog. Parallels can also be drawn between the *wt1a* expressing cells in the CP and testicular Sertoli cells, which are also polarized epithelial cells with tight junctions and are crucial for the blood-testis barrier. Wt1 is an established Sertoli cell marker and it has been found that *Wt1* inactivation in cultured mouse Sertoli cells results in loss of epithelial morphology and abnormal tight junction assembly (Wang et al., 2013). It is therefore tempting to speculate that similarly to its function in Sertoli cells and podocytes (Dong et al., 2015a), Wt1a is required for proper differentiation of the ependymal cells in the CP by orchestrating the expression of a number of genes that are essential for ependymal cell function.

However, there are also some important differences regarding *Wt1/wt1* expression in podocytes, Sertoli cells and ependymal cells. While *Wt1/wt1* is expressed in all developing and mature podocytes and Sertoli cells, *wt1a* is only expressed in a subpopulation of ependymal cells of the mCP. Also, we do not yet know whether Wt1a is present in the mCP of adult zebrafish. The *wt1a* expression in ependymal cells could either reflect a specific developmental state or could indicate the existence of different ependymal subpopulations. This has also been suggested by the use of two enhancer trap lines that labelled distinct subsets of mCP epithelial cells in zebrafish (Bill et al., 2008; Garcia-Lecea et al., 2008) and a very recent report using single cell RNA sequencing (D'Gama et al., 2021).

One possible signaling pathway that could be regulated by Wt1 in the context of choroid plexus formation and ependymal cell specification could be the Wnt/β-catenin pathway. The latter has been implicated in early stages of CP development in mice (Awatramani et al., 2003; Lun et al., 2015). Wt1 has been identified as a regulator of the Wnt/β-catenin pathway, although its precise role as an activator or inhibitor yet has to be defined (Kim et al., 2010; von Gise et al., 2011). Interestingly, it has been shown that downregulation of Wnt/β-catenin signaling leads to a hydrocephalus-like phenotype in mice (Ohtoshi, 2008; Del Bigio, 2010). We observe a similar phenotype in *wt1a* mutant zebrafish, although it remains to be determined whether this phenotypic similarity is caused by the same pathomechanism. On the other hand, it has been described that Wt1 mediates cell polarity and tight junction formation in bovine Sertoli cells via non-canonical Wnt signaling. By activating *Wnt4* expression, Wt1 regulates the key junctional protein occludin (Wang et al., 2019). Thus, possibly *wt1a* depletion causes downregulation of *occludin* expression, which might be responsible for the observed leakage of the mCP.

It has been pointed out that the molecular and cellular principles underlying mCP development in zebrafish are shared with other animals and thus very likely represent conserved mechanisms for CP development in vertebrates (Bill et al., 2008). On this background it will be interesting to explore whether *Wt1* is also expressed and has a function in the orthologous structures in mammals. In this context it is interesting to note that very recently a variant in the 3′-UTR of the human *WT1* gene has been reported in a patient with atypical choroid plexus papilloma tumor (Taher et al., 2019).

## Data Availability

The raw data supporting the conclusions of this article will be made available by the authors, without undue reservation.
