# Supplementary figures and images for: The Wilms Tumor Gene wt1a Contributes to Blood-Cerebrospinal Fluid Barrier Function in Zebrafish
Source: Front Cell Dev Biol. 2022 Jan 11;9:809962. doi: 10.3389/fcell.2021.809962 (PMC8786916; doi:10.3389/fcell.2021.809962)

**A**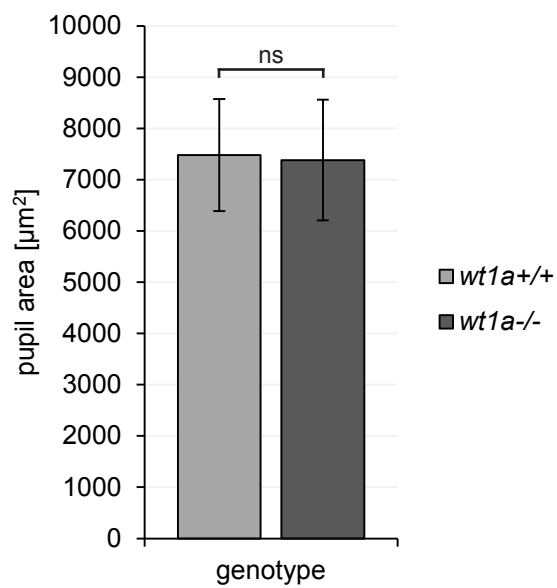**B**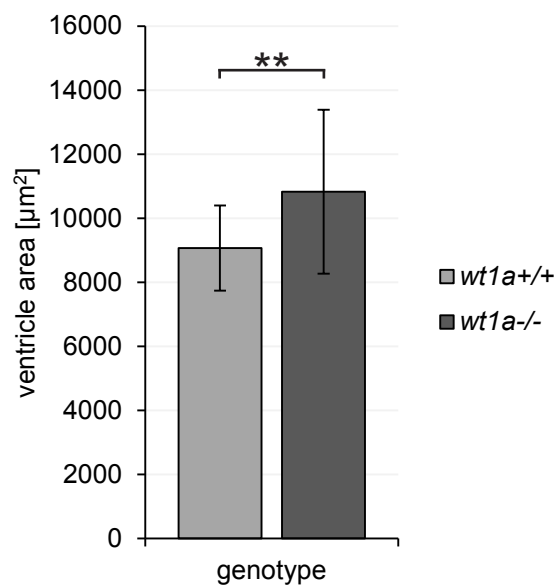

Supplement: Supplementary file 1 [file Image2.pdf]

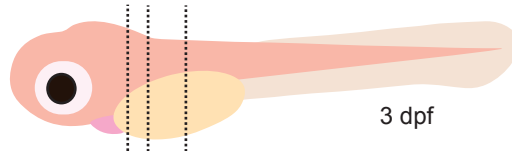

3 dpf

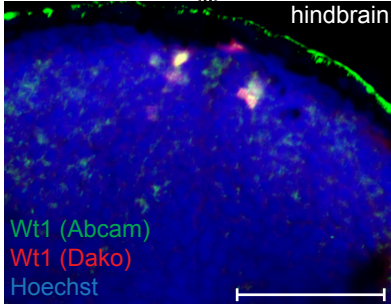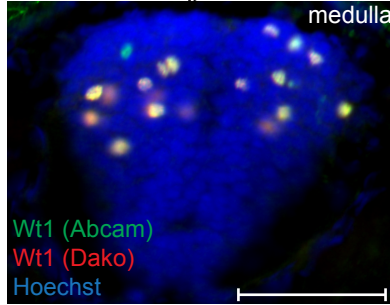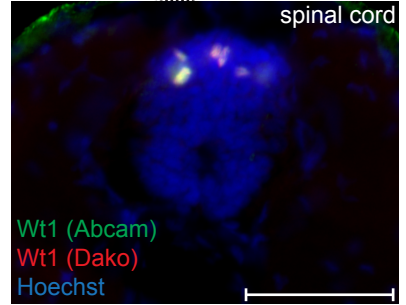

Supplement: Supplementary file 2 [file Image1.pdf]
